# Human CD8^+^ T cells transduced with an additional receptor bispecific for both *Mycobacterium tuberculosis* and HIV‐1 recognize both epitopes

**DOI:** 10.1111/jcmm.12878

**Published:** 2016-04-26

**Authors:** Chao‐Ying Zhou, Qian Wen, Xiao‐Jie Chen, Rui‐Ning Wang, Wen‐Ting He, Shi‐Meng Zhang, Xia‐Lin Du, Li Ma

**Affiliations:** ^1^ Institute of Molecular ImmunologySchool of BiotechnologySouthern Medical UniversityGuangzhouGuangdongChina

**Keywords:** *Mycobacterium tuberculosis*, human immunodeficiency virus type 1, coinfection, adoptive immunotherapy, T cell receptor, CD8^+^ T cells, cross‐reactive

## Abstract

Tuberculosis (TB) and human immunodeficiency virus type 1 (HIV‐1) infection are closely intertwined, with one‐quarter of TB/HIV coinfected deaths among people died of TB. Effector CD8^+^ T cells play a crucial role in the control of *Mycobacterium tuberculosis* (MTB) and HIV‐1 infection in coinfected patients. Adoptive transfer of a multitude of effector CD8^+^ T cells is an appealing strategy to impose improved anti‐MTB/HIV‐1 activity onto coinfected individuals. Due to extensive existence of heterologous immunity, that is, T cells cross‐reactive with peptides encoded by related or even very dissimilar pathogens, it is reasonable to find a single T cell receptor (TCR) recognizing both MTB and HIV‐1 antigenic peptides. In this study, a single TCR specific for both MTB Ag85B_199‐207_ peptide and HIV‐1 Env_120‐128_ peptide was screened out from peripheral blood mononuclear cells of a HLA‐A*0201^+^ healthy individual using complementarity determining region 3 spectratype analysis and transferred to primary CD8^+^ T cells using a recombinant retroviral vector. The bispecificity of the TCR gene‐modified CD8^+^ T cells was demonstrated by elevated secretion of interferon‐γ, tumour necrosis factor‐α, granzyme B and specific cytolytic activity after antigen presentation of either Ag85B_199‐207_ or Env_120‐128_ by autologous dendritic cells. To the best of our knowledge, this study is the first report proposing to produce responses against two dissimilar antigenic peptides of MTB and HIV‐1 simultaneously by transfecting CD8^+^ T cells with a single TCR. Taken together, T cells transduced with the additional bispecific TCR might be a useful strategy in immunotherapy for MTB/HIV‐1 coinfected individuals.

## Introduction

Tuberculosis (TB) and human immunodeficiency virus (HIV) infections are the world's deadliest chronic contagion. Tuberculosis is a major cause of morbidity and mortality in HIV‐infected individuals and is more likely to become disseminated among these people. It has been reported by the World Health Organization that an estimated 1.1 million (13%) of the 9 million new TB cases in 2013 were coinfected with HIV; in total, approximately 1.5 million people died from TB, around 1/4 of who were HIV positive 1. In the host, *Mycobacterium tuberculosis* (MTB) and HIV potentiate each other, accelerating the deterioration of immunological functions 2. Once individuals with latent TB infection (LTBI) are infected by HIV, the destruction of the immune system will be accelerated with regard to a decline in function and number of CD4^+^ T cells. The destroyed immune system cannot inhibit MTB anymore, and the LTBI persons are easier to develop active TB 3, 4. Meanwhile, MTB stimulates monocytes and macrophages to secrete great number of monocyte chemotactic protein‐1, which promotes disease progression by facilitating HIV transcription and virus proliferation 5. Currently, the treatment of MTB/HIV coinfection by combining isoniazid preventive therapy and antiretroviral therapy (ART) had certain curative effects but raised multiple problems, including long course of treatment, potential drug interactions 6, overlapping toxicity profiles 7, a high pill burden, programmatic challenges 8, immune reconstitution inflammatory syndrome 9, *etc*. Therefore, a safer, more effective and accessible treatment of MTB/HIV coinfection is urgently needed.

The main immune mechanism of anti‐MTB/HIV coinfection is cellular immunity mediated by T cells. Considerable experimental evidence implicated that antigen‐specific CD8^+^ T cells play a crucial role in the control of both MTB and HIV infection 10, 11. Effector CD8^+^ T cells are necessary to kill intracellular pathogens by producing the T helper type 1 cytokines interferon‐γ (IFN‐γ) and tumour necrosis factor‐α (TNF‐α), which activate the mycobactericidal mechanisms of macrophages 12. Furthermore, CD8^+^ T cells exert direct cytotoxicity on infected target cells *via* releasing perforin and granzyme proteases 12. However, upon the condition of MTB/HIV‐1‐coinfection, whole disfunction of cellular immunity is unavoidable 13, 14. Targeting this problem, the most convenient and effective way is adoptive transfer of vast numbers of active effector CD8^+^ T cells to coinfected individuals.

Adoptive cellular immunotherapy has shown great potential in anti‐MTB and anti‐HIV infection. For patients with multidrug‐resistant TB, infusion of peripheral blood lymphocytes stimulated with inactivated MTB *ex vivo* achieved excellent curative effects 15. Lieberman *et al*. 16 infused autologous CD8^+^ cytotoxic T cells to six HIV‐seropositive patients and observed increase in CD4^+^ T cell numbers in all patients and decrease in plasma viremia in five of six patients during the first 2 weeks. Twenty‐four weeks later, cell‐associated viral burden in two patients continued decreasing and another patient had more than doubled CD4^+^ T cell count. Although showing a bright future, there are still many obstacles in universal application of adoptive cellular immunotherapy. It is usually difficult to isolate sufficient numbers of effector CD8^+^ T cells with defined specificity, caused by either exhausted cytotoxic T lymphocyte (CTL) responses due to advanced disease 14, 17 or frequency fall‐off of pathogen‐reacting CTL precursors following pathogen burden reduction in individuals receiving long‐term treatment 18. In addition, terminal differentiation of T cells isolated from infected individuals often limits their function 17. Difficulty in expansion *ex vivo* and long‐term maintenance *in vivo* after infusion are also obstacles. However, these problems can be effectively solved with transferring antigen‐specific T cell receptor (TCR) gene‐modified T cells, which makes the heterogenous T cells recognize the specific antigen artificially and plenty of effector T cells can be obtained in short term 19.

Our previous work proved improved functional avidity of engineered CD4^+^ and CD8^+^ T cells with MTB 38‐kD antigen‐specific TCRs 20. Both *in vitro* and *in vivo* excellent effects of gene modification of CD8^+^ T cells with specific TCR targeting the HIV‐1 gag epitope have also been reported 21. However, modification of T cells with one single TCR gene simultaneously targeting both antigens of MTB and HIV‐1 has never been reported, while it is consistent with the theory of T cell cross‐reactivity.

In humans, researchers estimated that there are <10^8^ distinct TCRs in the naïve T cell pool 22, which is dwarfed by a substantial number of potential foreign peptide‐MHC complexes (>10^15^ distinct peptide‐MHCs) 23. Consequently, adaptive T cell immunity requires each T cell to recognize a multitude of potential antigen peptides, as demonstrated by the phenomenon of T cell cross‐reactivity 24. One excellent example is the recently described 1E6 TCR isolating from a patient with type 1 diabetes. Besides recognizing the preproinsulin‐derived HLA‐A*0201‐restricted peptide PPI_15‐24_ (ALWGPDPAAA) 25, T cells expressing the 1E6 TCR could respond to over 1.3 million 10‐mer peptides at least as strongly as they respond to the PPI_15‐24_ peptide 26, 27. Among these huge number of peptide, the RQFGPDFPTI (sampled from >10^8^ peptides) was >100‐fold more potent than PPI_15‐24_ at activating 1E6 TCR‐expressing T cells despite differing from PPI_15‐24_ at 70% of amino acid (AA) composition 27. Therefore, it is absolutely reasonable to find a single TCR recognizing both two antigen peptides.

Here, we generated the bifunctional T cell population by introduction of a bispecific TCR by means of retroviral transfer. These T cells are capable of recognizing both HLA‐A*0201‐restricted MTB Ag85B_199‐207_ (KLVANNTRL) and HLA‐A*0201‐restricted HIV‐1 Env_120‐128_ (KLTPLCVTL) peptides. We demonstrated the presence of both anti‐MTB and anti‐HIV‐1 reactivity in TCR‐transferred dual‐specific T cells *in vitro*. To the best of our knowledge, this is the first time that human T cells have been artificially equipped with one bispecific TCR specific for both MTB and HIV‐1 antigen peptides. This study provided a promising strategy in immunotherapy for MTB/HIV coinfected patients.

## Materials and methods

### Isolation and culture of peptide‐specific T cells

The protocol was approved by the ethics committee of the Southern Medical University. Peripheral blood mononuclear cells (PBMCs) were isolated from whole blood of a HLA‐A*0201 healthy donor with informed consent by Ficoll‐Hypaque (Shanghai Second Chemistry Factory, Shanghai, China) density gradient centrifugation. Peripheral blood mononuclear cells were cultured in 6‐well plates (Nunc, Roskilde, Denmark) in RPMI‐1640 medium (Hyclone Ltd., Logan, UT, USA) contained with 10% foetal bovine serum (FBS; Hyclone Ltd.) and 100 U/ml interleukin‐2 (IL‐2; PeproTech, Rocky Hill, NJ, USA). Monocyte‐derived dendritic cells (DCs) 28 induced from the autologous PBMCs were loaded with 50 μg/ml of HLA‐A*0201‐restricted peptides MTB Ag85B_199–207_ (KLVANNTRL) or HIV‐1 Env_120‐128_ (KLTPLCVTL) (both from Beijing AuGCT DNA‐SYN Biotechnology Co., Ltd, Beijing, China) for 2–4 hrs at 37°C and then used as antigen‐presenting cells to coculture with autologous PBMCs at a stimulator to responder cell ratio of 1:10. The responding PBMCs were restimulated twice with peptide‐pulsed DCs at weekly intervals in the presence of 100 U/ml IL‐2. Following the second restimulation, PBMCs were collected for CD8^+^ T cell sorting.

### Sorting of CD8^+^ T cells using magnetic beads

CD8^+^ T cells were sorted from Ag85B_199‐207_‐ and Env_120‐128_‐stimulated PBMCs using anti‐CD8‐labelled MACS magnetic beads (Miltenyi Biotec, Bergisch Gladbach, Germany) as per the instruction of the manufacturer.

### Isolation of peptide‐specific TCRs from primed T cells

GeneScan analysis of TCR complementarity determining region 3 (CDR3) spectratype was performed as previously described 29. Briefly, RNA was extracted from Ag85B_199–207_‐ and Env_120‐128_‐stimulated CD8^+^ T cells using E.Z.N.A.^®^ Total RNA Kit (OMEGA Biotek, Inc., Norcross, GA, USA) according to the manufacturer's instruction, and reverse transcription was performed using RevertAid^™^ First Strand cDNA Synthesis Kit (Fermentas, Life Sciences, Burlington, Ontario, Canada). The TCR of 34 α‐chain variable region (Vα) and 24 β‐chain variable region (Vβ) gene families were amplified. Antigen‐specific Vα or Vβ gene family was determined by CDR3 spectratype analysis.

### Construction of the retroviral vector

According to the antigen‐specific CD8^+^ TCR Vα and Vβ gene family identified by TCR CDR3 spectratype analysis, primers were designed to amplify the full‐length TCR α17 (accession no.: NG_001332.2) and β15 (accession no.: NG_001333.2) coding sequences using recombinant PCR methods. Nine AAs in the constant regions were replaced by murine counterparts as described previously 30.

Briefly, to achieve TCR β15‐chain, the peptide‐stimulated CD8^+^ T cells cDNA was firstly used as the template. The forward primer P1 and the reverse primer P6 (containing 5′‐end of P2A) were used to generate the wild‐type β‐chain containing 5′‐end of P2A. Using this product as the template, the primers P1 and P2 (containing the 2‐AA mutation near to the amino‐terminal of constant region) were used to generate Vβ15 containing the 2‐AA mutation (hereinafter referred to as S1). Meanwhile, the primers P3 (containing the same 2‐AA mutation) and P4 (containing the 3‐AA mutation near to the carboxyl‐terminal of constant region) were used to generate the amino‐terminal of Cβ containing the 5‐AA mutation (fragment S2). The primers P5 (containing the same 3‐AA mutation) and P6 were used to generate the carboxyl‐terminal of Cβ containing both the 3‐AA mutation and 5′‐end of P2A (fragment S3). All of above reactions constitute the first round PCR. Using S1 and S2 as the templates, a second PCR using P1 and P4 generated the fragment of Vβ15 containing the 5‐AA mutation (fragment S4). Using S3 and S4 as the templates, a third PCR using P1 and P6 completed the β‐chain. Similarly, to generate TCR α17‐chain, with the peptide‐stimulated CD8^+^ T cells cDNA as the template, the forward primer P7 (containing 3′‐end of P2A) with the reverse primer P10 was used to generate the wild‐type α‐chain containing 3′‐end of P2A. Using this product as the template, the primers P7 and P8 (containing the 4‐AA mutation) were used to generate Vα17 containing the 4‐AA mutation (fragment S5). At the same time, the primers P9 (containing the same 4‐AA mutation) and P10 were used to generate Cα containing the 4‐AA mutation (fragment S6). Then, using S5 and S6 as the templates, primers P7 and P10 completed the α‐chain. The full‐length TCR β15‐P2A‐α17 was formed using the β15 and α17 products as the templates and with the primers P1 and P10.

The TCR β15‐P2A‐α17 product was digested and inserted into the pMX‐internal ribosomal entry site (IRES)‐green fluorescent protein (GFP) retroviral vector (kindly provided by Han H, Fourth Military Medical University, Xi'an, China) at BamH I and Xho I sites to construct the pMX‐β15‐P2A‐α17‐IRES‐GFP recombinant retroviral vector. To increase translation efficiency, a Kozak consensus sequence 31 was added into the primer P1. A GSG (Gly‐Ser‐Gly) spacer 32 was incorporated into the primer P6 to ensure maximal TCR β: TCR α cleavage. All of the primers (P1‐P10) used to generate TCR β15‐P2A‐α17 are outlined in Table 1.

**Table 1:**
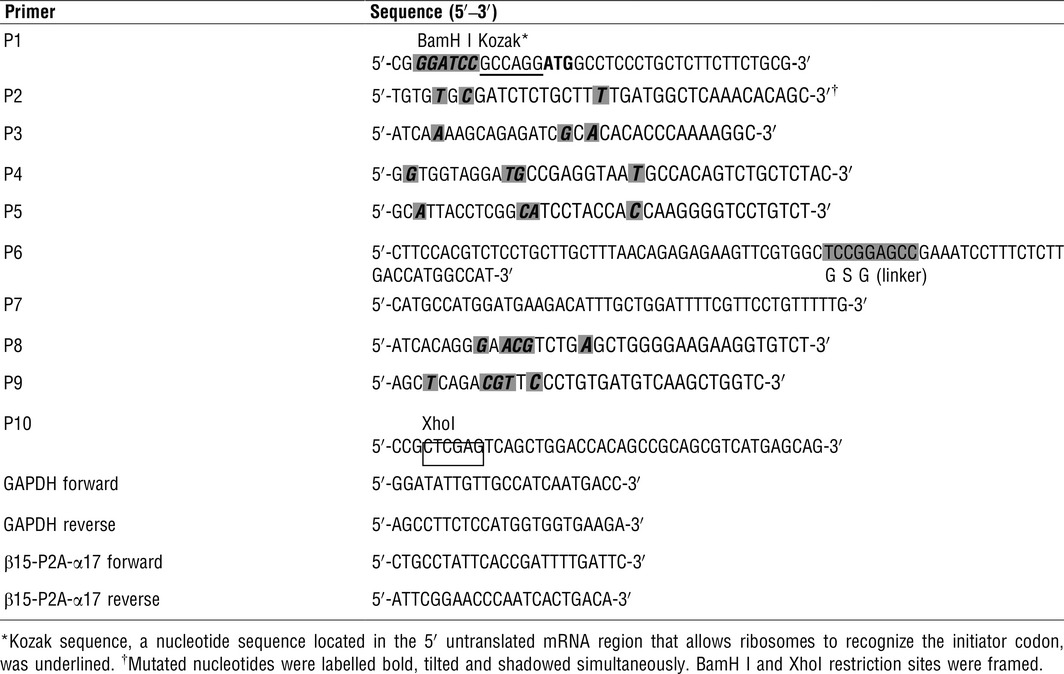
Primers used for amplification of TCR β15‐P2A‐α17 and employed in quantitative real‐time RT‐PCR

### Retrovirus production

The recombinant vector and the VSV‐G envelope protein vector were cotransfected into the GP2‐293 packaging cells 33 using Lipofectamine 2000 Transduction Reagent (Invitrogen, Carlsbad, CA, USA) following the manufacturer's instructions. Viral supernatants were harvested 48–72 hrs later and concentrated by ultracentrifugation at 50,000 × *g*, 4°C, for 90 min., using a high‐speed refrigerated centrifuge (Hitachi Koki Co., Ltd. Kyoto, Japan). The recombinant retroviral particles were resuspended in fresh serum‐free RPMI‐1640 medium at 1% volume of the original culture supernatant and stored at −70°C.

To determine the viral titres, NIH3T3 cells were plated at 1 × 10^6^ cells in 1 ml of 10% FBS DMEM medium (Hyclone Ltd.) per well in 6‐well plates 24 hrs before infection. Then, 5 μl of the concentrated virus suspension containing polybrene with final concentration of 8 μg/ml/well (Sigma‐Aldrich, St Louis, MO, USA) was added. The culture supernatants were replaced with fresh culture medium 24 hrs after infection and viral titres measured by flow cytometry 3 days after infection. The titre was calculated as follows: GFP‐positive rate × 10^6^ cells/5 μl, expressed as infectious units per millilitre (IU/ml).

### Transduction of CD8^+^ T cells

CD8^+^ T cells were inoculated at 1 × 10^6^ cells/ml in 6‐well plates in the presence of 100 U/ml IL‐2 and 50 ng/ml OKT3 antibody (Ortho Biotech, Raritan, NJ, USA) 72 hrs before transduction. The concentrated recombinant virus suspension containing 8 μg/ml of polybrene (according to the final media volume) was added, and 4 hrs later the fresh medium was supplemented to dilute the polybrene to 2 μg/ml. Five days after transduction, gene‐modified CD8^+^ T cells were collected to detect the expression of GFP and incubated with PE‐Cy7‐anti‐CD8 (eBioscience, San Diego, CA, USA), PE‐labelled Ag85B_199‐207_/HLA‐A*0201 dextramer and APC‐labelled Env_120‐128_/HLA‐A*0201 dextramer (Immudex, Copenhagen, Denmark) according to the manufacturer's instructions to detected the exogenous TCR by flow cytometry.

### Real‐time quantitative PCR analysis

The FastStart Universal SYBR Green Master (ROX) kit (Roche Applied Science, Mannheim, Germany) and ABI PRISM 7900HT Sequence Detection System (Applied Biosystems Inc., Foster City, CA, USA) were used to perform qRT‐PCR. Quantification of target mRNA abundance was analysed with the SDS software version 2.3 (Applied Biosystems Inc.) using the comparative threshold cycle (Ct) method 34. Relative quantification relates the PCR signal of the sample transcript in the transduced CD8^+^ T cells to that of the control CD8^+^ T cells at each time. The fold change in cDNAs (target gene) after GAPDH (reference gene) normalization was determined by the following formula: fold change = 2^−ΔΔCt^, where ΔΔCt = (Ct_Target_ − Ct_GAPDH_)_sample_ − (Ct_Target_ − Ct_GAPDH_)_control_. Primer sequences used to amplify GAPDH and β15‐P2A‐α17 from human CD8^+^ T cells are summarized in Table 1.

### Cytokine assays

To determine the function of the TCR gene‐modified T cells upon antigen stimulation, experiment groups were set up as indicated in figure legends. Peptide (10 μg/ml, or as described in figure legends)‐loaded or peptide‐unloaded autologous DCs were seeded at 5 × 10^3^ cells/well in a 96‐well plate (Nunc) and cocultured with CD8^+^ T cells according to the different effector: target (E:T) ratio, 7 in IFN‐γ assays and 20 in TNF‐α and granzyme B (GrB) assays, respectively. In some groups, DCs were transfected with the pV1J.ns‐tPA‐Ag85B plasmid (gifted by Dr. Kris Huygen in Pasteur Institute of Brussels, Brussels, Belgium), the pCAGGS‐Env plasmid (gifted by Dr. James M. Binley in Torrey Pines Institute for Molecular Studies, San Diego, CA, USA) or the pCI‐OVA plasmid (kindly provided by Dr. Yukio Koide, Hamamatsu University School of Medicine, Hamamatsu, Japan), respectively, using Lipofectamine 2000 Transduction Reagent (Invitrogen). The culture supernatants were harvested 18 hrs later for determining IFN‐γ levels and 24 hrs later for TNF‐α and GrB. Cytokines were measured using IFN‐γ, TNF‐α ELISA kits (Bender MedSystems, Vienna, Austria) and GrB ELISA Kit (R&D Systems, Inc., Minneapolis, MN, USA) according to the manufacture's protocols. Both E:T ratios and incubation time were determined according to our previous study 20.

### Cytotoxicity assays

Cytotoxicity of transduced T cells was measured using a DELFIA EuTDA cytotoxicity kit (Perkin‐Elmer Life Sciences, Norwalk, CT, USA) according to the manufacture's instruction. Groups were set up same as described above. Eu‐labelled autologous DCs (5 × 10^3^) were cocultured with TCR‐transduced CD8^+^ T cells at the E:T ratio of 30:1. Four hours later, supernatants were collected to detect the cytolytic activity. Fluorescence was measured using a Wallac Victor 2 Multilabel Counter (Perkin‐Elmer Life Sciences). The % specific lysis was determined using the following formula: [(experimental release − spontaneous release)/(maximum release − spontaneous release)] × 100, where the spontaneous release was detected by reading the values of the target cells without effector cells, and the maximum release was detected by completely lysing labelled target cells.

### Intracellular cytokine staining

For intracellular IFN‐γ staining, TCR gene‐modified CD8^+^ T cells were stimulated by Ag85B_199‐207_ or Env_120‐128_ peptide‐loaded DCs in the presence of IL‐2 (50 U/ml) and Brefeldin A (10 μg/ml, Sigma). After 24 hrs, 1 × 10^6^ of cocultured cells were centrifugated at 300 × *g* for 4 min. and washed by 5% FBS‐PBS for 1–2 times, then fixed and permeabilized with BD Cytofix/Cytoperm^™^ Fixation/Permeabilization Solution Kit (BD Pharmingen Company, San Jose, CA, USA) and stained with PE‐Cy7‐anti‐CD8 and PE‐anti‐IFN‐γ (eBioscience) according to the instructions. Data acquisition and analysis was done by flow cytometry.

### CD69 expression in transduced J.RT3‐T3.5 cells

The concentrated recombinant virus suspension was used for infecting the TCR β‐chain‐deficient Jurkat T cell line J.RT3‐T3.5 (kindly provided by Dr. Wei He, Peking Union Medical College, Beijing, China). Three days after transduction, gene‐modified J.RT3‐T3.5 cells were collected and cocultured with T2 cells that had been loaded with peptide (10 μg/ml, or as described in figure legends). After 18 hrs, the cocultured cells were collected, centrifuged at 300 × *g* for 5 min., washed by 5% FBS‐PBS for 1–2 times and then stained with APC‐anti‐CD69 (eBioscience) according to the instructions. Data acquisition and analysis was done by flow cytometry.

### Statistical analysis

All statistical analyses were performed using the SPSS version 17.0 for windows (SPSS, Chicago, IL, USA). A one‐way anova and multiple comparisons tests (least significant difference or Dunnett's T3) were used to compare the differences between the experimental groups. The level of significance used was *P* < 0.05. *P*‐values were two‐sided.

## Results

### Screening for a single TCR specific for both MTB and HIV‐1 peptide

**Figure 1:**
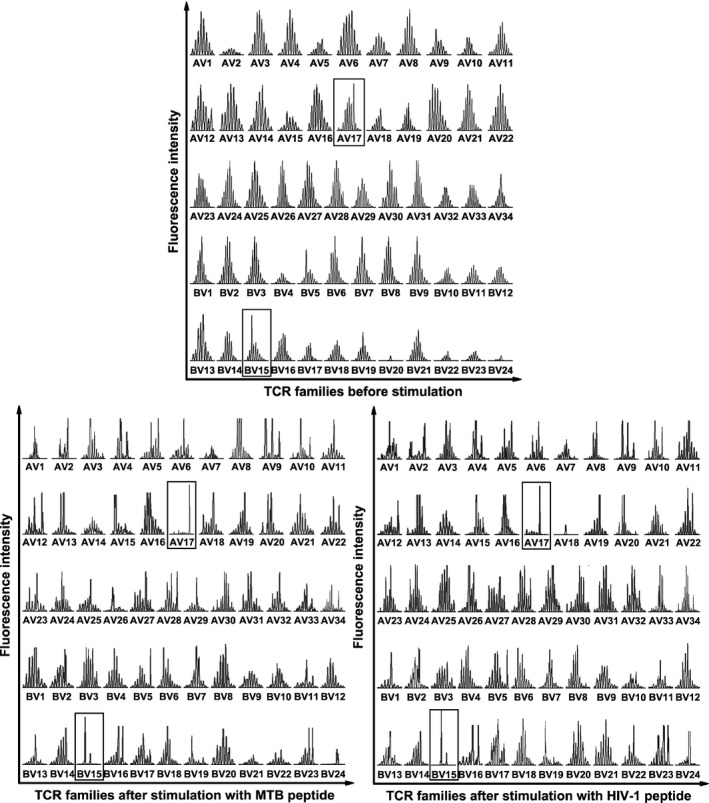
CDR3 spectratypes of 34 TCR Vα and 24 Vβ gene families of CD8^+^ T cells before and after antigen peptide stimulation. Both spectratypes of the MTB Ag85B_199‐207_ (KLVANNTRL) peptide‐stimulated CD8^+^ T cells and of the HIV‐1 Env_120‐128_ (KLTPLCVTL) peptide‐stimulated CD8^+^ T cells showed single peaks in TCR Vα17 and Vβ15 gene families (framed) after stimulation, indicating its specificity for corresponding peptides. AV, TCR Vα‐chain; BV, TCR Vβ‐chain.

In this study, CDR3 spectratype analysis showed that the TCR repertoire of unstimulated T cells exhibited a Gaussian distribution with eight peaks or more. In contrast, following peptide stimulation, part of the TCR families showed skewed distribution as indicated by oligo‐peak or even single‐peak. The unimodality of TCR family distribution resulted from monoclonal expansion of T cells after antigen stimulation and indicated that the corresponding gene families were peptide specific. In the spectratyping data, more than one TCR Vα and Vβ gene families have changed after both MTB and HIV‐1 peptides stimulation, *e.g*. AV6, 8, 9 and 17; and BV15, 16 and 24. But only AV17 and BV15 simultaneously exhibited single‐peak after the two peptides stimulation, while other gene families exhibited oligo‐peak. Moreover, the relative fluorescence intensity of single‐peak in AV17 and BV15 is the highest compared with other AV and BV gene families, respectively, demonstrating that the corresponding monoclonal T lymphocytes may have better activity against antigenic peptides. Hence, the most represented gene families AV17 and BV15 were supposed to be MTB Ag85B_199–207_ and HIV‐1 Env_120‐128_ specific (Fig. 1).

### Construction of the retroviral vector carrying MTB/HIV bispecific TCR gene

**Figure 2:**
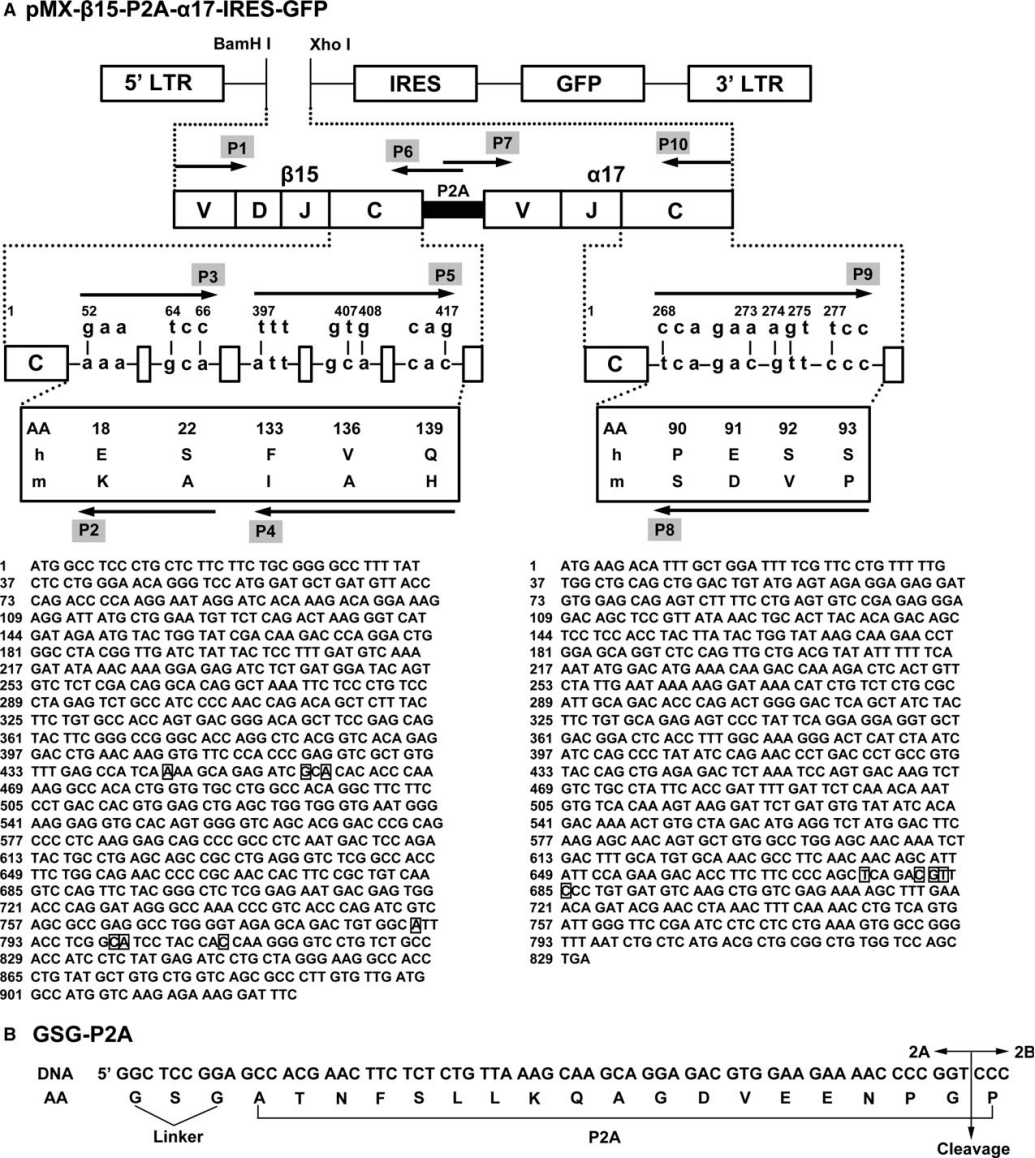
Construction of a retroviral vector expressing MTB Ag85B_199‐207_ and HIV‐1 Env_120‐128_‐bispecific TCR genes. (**A**) Schematic representation of retroviral expression vector (pMX‐IRES‐GFP) and P2A‐linked TCR fusion gene (β15‐P2A‐α17). Nine critical amino acids in the constant regions (C) of β15 and α17 were replaced by their murine counterparts. TCR β‐chain and α‐chain were linked by P2A peptide. The sequences of TCR β15 (the lower left panel) and α17 (the lower right panel) were displayed. (**B**) Details of GSG‐P2A sequences. A GSG flexible linker was incorporated between the TCR β‐chain and the P2A peptide. The P2A cleavage point between the 2A and 2B peptides, and thus the N‐ and C‐terminal cistrons, is indicated by the arrow. LTR: long terminal repeat; IRES: internal ribosomal entry site; GFP: green fluorescent protein; AA: the amino acid sequence; h: human; m: murine; DNA: the DNA sequence.

In order to promote preferential pairing and increase total surface expression of the introduced TCR α‐ and β‐chains, we replaced nine critical AAs in the human TCRα and TCRβ constant regions by their murine counterparts using recombinant PCR 30. In the meantime, P2A was used to link the TCR α‐ and β‐ chains, which ensures their equimolar production *via* a ‘ribosomal skip’ mechanism to generate two proteins from one mRNA chain, one containing N‐terminal of 2A (2A peptide) and another containing C‐terminal of 2A (2B peptide) 35. A GSG linker ensuring complete ‘cleavage’ between the TCR β‐chain and the P2A peptide was added (GSG‐P2A). After that, the mouse–human hybrid construct of the full‐length TCR β15‐P2A‐α17 gene was cloned into a retroviral vector to obtain the recombinant vector pMX‐β15‐P2A‐α17‐IRES‐GFP (Fig. 2). The recombinant vector and the VSV‐G envelope protein vector were cotransfected into the GP2‐293 packaging cells. Then, viral supernatants were harvested and concentrated. The infectious viral titre was 1.08 × 10^8^ IU/ml.

### Expression of exogenous TCR genes

**Figure 3:**
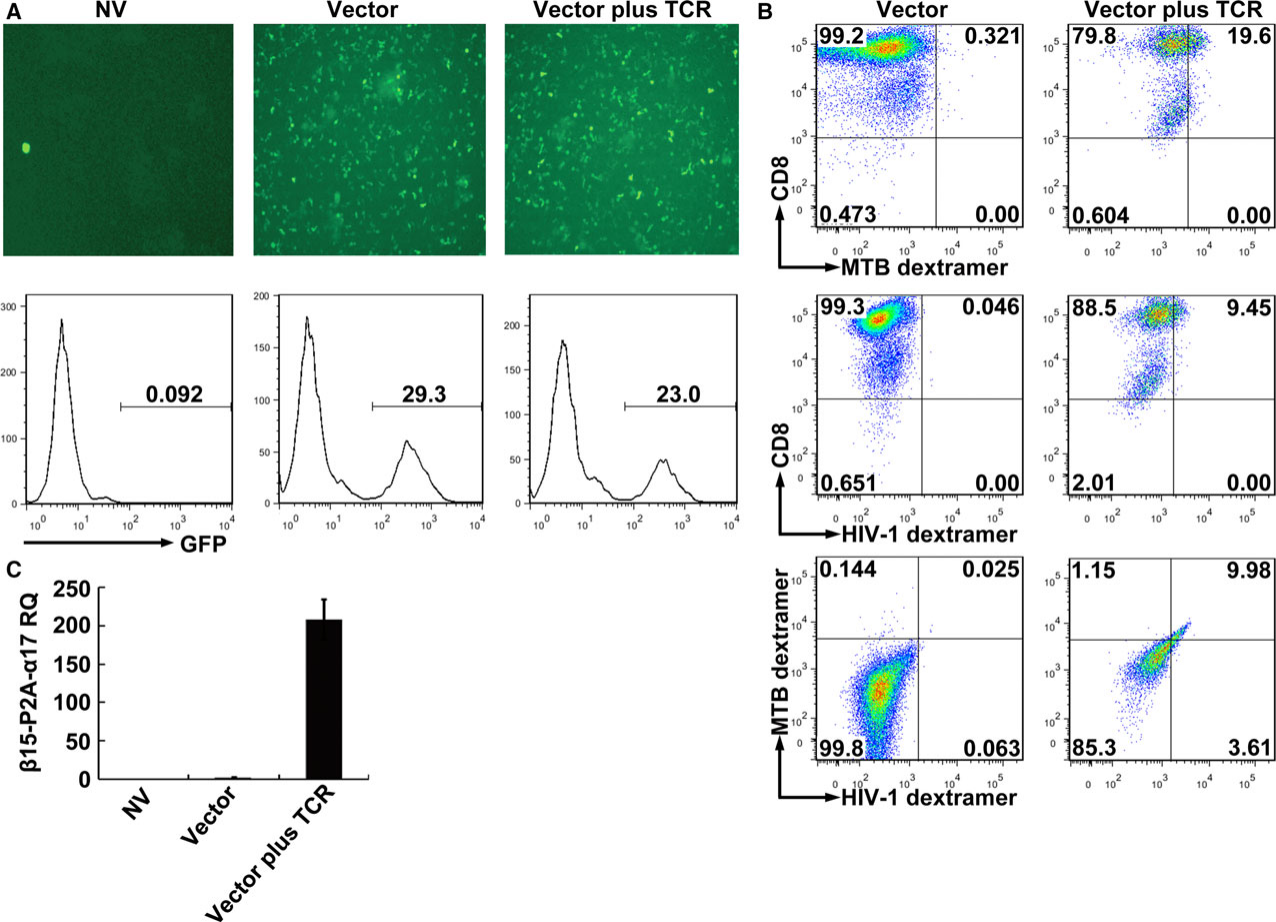
Expression of exogenous TCRs after genetic transferring TCR genes into CD8^+^ T cells. (**A**) GFP expression was observed under fluorescence microscope and analysed by FACS. (**B**) Flow cytometric analysis of CD8^+^ T cells 5 days after retroviral transduction. Cells were stained with PE‐Cy7‐anti‐CD8, PE‐Ag85B_199‐207_/HLA‐A*0201 dextramer and APC‐Env_120‐128_/HLA‐A*0201 dextramer. The data were analysed within the GFP‐positive population. (**C**) Quantitative RT‐PCR analysis of mRNA expression of β15‐P2A‐α17 genes. NV, transduced with no vector; Vector, transduced with empty vector only carrying the GFP gene; Vector plus TCR, transduced with vector carrying the GFP gene and MTB Ag85B_199‐207_‐ and HIV‐1 Env_120‐128_‐bispecific TCR gene; MTB dextramer: PE‐Ag85B_199‐207_/HLA‐A*0201 dextramer; HIV‐1 dextramer: APC‐Env_120‐128_/HLA‐A*0201 dextramer; RQ: relative mRNA expression normalized to GAPDH.

Five days after transduction, expression of GFP reporter gene was clearly observed in TCR gene‐modified CD8^+^ T cells but not in untransduced CD8^+^ T cells under the fluorescence microscope (Fig. 3A, upper panel). Further measurement by flow cytometry showed that the percentage of the GFP‐positive CD8^+^ T cells after transduction was more than 20%, while no definite fluorescence signal was detected in untransduced cells (Fig. 3A, lower panel). As there is presently no anti‐TCR Vα17 or Vβ15 antibody commercially available, we used MHC dextramers to detect the expression of exogenous TCR. As determined by MHC dextramer flow cytometry, about 10% of the total CD8^+^ T cells were positive for the Ag85B_199‐207_/HLA‐A*0201 dextramer and Env_120‐128_/HLA‐A*0201 dextramer simultaneously within the GFP‐positive population, which demonstrated the successful expression of introduced bispecific TCR in CD8^+^ T cells. In contrast, only very low background staining was observed in empty vector‐transduced cultures (Fig. 3B). Transcription of exogenous TCR genes was further verified by qRT‐PCR. The expression of transferred TCR genes was significantly higher in the TCR gene‐modified cells than in the empty vector‐transduced cells and the untransduced cells (Fig. 3C).

### Peptide‐specific IFN‐γ and TNF‐α secretion in TCR gene‐modified CD8^+^ T cells

**Figure 4:**
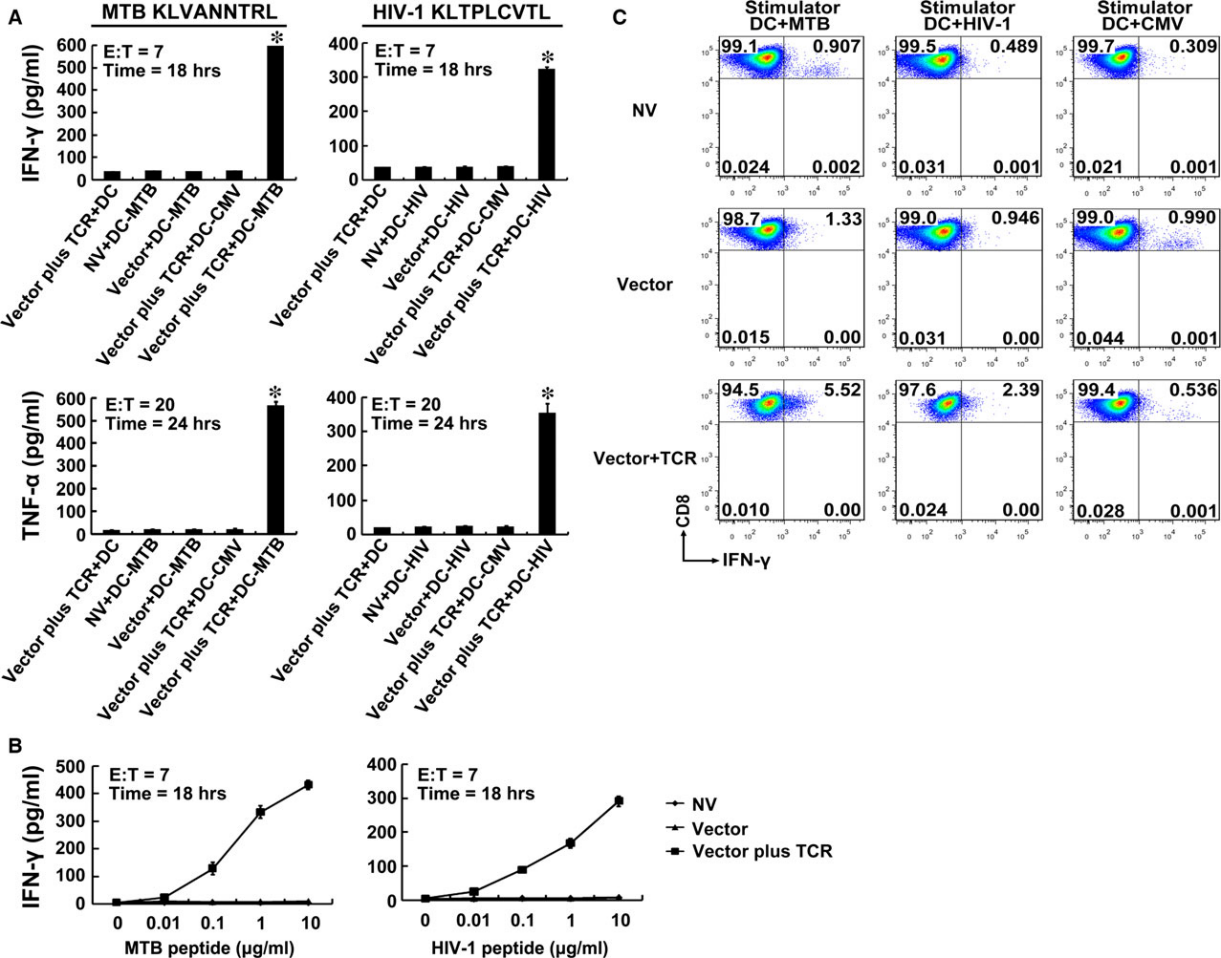
Cytokine secretion of TCR gene‐modified CD8^+^ T cells in response to antigen peptides stimulation. (**A**) CD8^+^ T cells were cocultured with DCs loaded with 10 μg/ml of MTB Ag85B_199‐207_ (KLVANNTRL) (left panel) or HIV‐1 Env_120‐128_ (KLTPLCVTL) (right panel). Data represent average values of three independent experiments ± SD. *P < 0.05 compared with NV + DC‐MTB group or NV + DC‐HIV group. Vector plus TCR + DC: TCR gene‐modified CD8^+^ T cells + DCs unloaded with peptide; NV + DC‐MTB: CD8^+^ T cells transduced with no vector + MTB Ag85B_199‐207_‐loaded DCs; NV + DC‐HIV: CD8^+^ T cells transduced with no vector + HIV‐1 Env_120‐128_‐loaded DCs; Vector + DC‐MTB: empty vector‐transduced CD8^+^ T cells + MTB Ag85B_199‐207_‐loaded DCs; Vector + DC‐HIV: empty vector‐transduced CD8^+^ T cells + HIV‐1 Env_120‐128_‐loaded DCs; Vector plus TCR + DC‐CMV: TCR gene‐modified CD8^+^ T cells + CMV pp65_495‐503_ (NLVPMVATV)‐loaded DCs; Vector plus TCR + DC‐MTB: TCR gene‐modified CD8^+^ T cells + MTB Ag85B_199‐207_‐loaded DCs; Vector plus TCR + DC‐HIV: TCR gene‐modified CD8^+^ T cells + HIV‐1 Env_120‐128_‐loaded DCs. (**B**) Sensitivity of cytokine secretion to dilutions of Ag85B_199‐207_ or Env_120‐128_ peptide. MTB peptide: Ag85B_199‐207_ peptide; HIV‐1 peptide: Env_120‐128_ peptide. (**C**) Intracellular cytokine staining of transduced CD8^+^ T cells. Shown are the resultant FACS scatter plots for CD8^+^ T cells transduced with no vector, empty vector or TCR vector. Cells were cocultured with DCs pulsed with Ag85B_199‐207_, Env_120‐128_ or control peptide CMV pp65_495‐503_.

T cell receptor gene‐modified CD8^+^ T cells stimulated by MTB Ag85B_199‐207_‐loaded DCs produced significantly higher levels of IFN‐γ (*P* < 0.01) and TNF‐α (*P* < 0.01) than untransduced or empty vector‐transduced T cells, suggesting that these T cells were endowed with enhanced activity after TCR gene modification. Meanwhile, compared with untransduced group, TCR gene‐modified CD8^+^ T cells cocultured with DCs loaded with unassociated CMV pp65_495‐503_ peptide did not show any increase in activation (*P* > 0.05), indicating that the TCR gene‐modified CD8^+^ T cells responded specifically to MTB Ag85B_199‐207_ stimulation (Fig. 4A, left panel).

Similarly, the TCR gene‐modified T cells also showed a significant increase in cytokine secretion only after HIV‐1 Env_120‐128_ peptide stimulation compared with control groups (*P* < 0.01). These data demonstrated that the improved function following TCR gene modification was also Env_120‐128_ peptide specific (Fig. 4A, right panel).

The relative avidity of the TCR gene‐engineered T cells was determined by coculturing transduced CD8^+^ T cells with DCs pulsed with serial dilutions of the Ag85B_199‐207_ or Env_120‐128_ peptide (Fig. 4B). Responses of the modified CD8^+^ T cells to both peptides were activated at peptide concentrations as low as 0.01 μg/ml and increased in a dose‐dependent manner. In contrast, no significant differences in cytokine production were observed in untransduced and empty vector‐transduced cell populations at every peptide concentrations.

Intracellular cytokine staining revealed that more than 5% and 2% of the TCR gene‐modified CD8^+^ T cells within the whole population of transduced T cells were double positive for CD8 and intracellular IFN‐γ after Ag85B_199‐207_ peptide or Env_120‐128_ peptide presentation by DCs, respectively. In contrast, only a very low proportion of double‐positive T cells was observed in the untransduced or empty vector‐transduced groups. These results further indicated that the TCR gene‐modified CD8^+^ T cells responded specifically to both MTB Ag85B_199‐207_ and HIV‐1 Env_120‐128_ stimulation (Fig. 4C).

### Cytotoxicity of TCR gene‐modified CD8^+^ T cells

**Figure 5:**
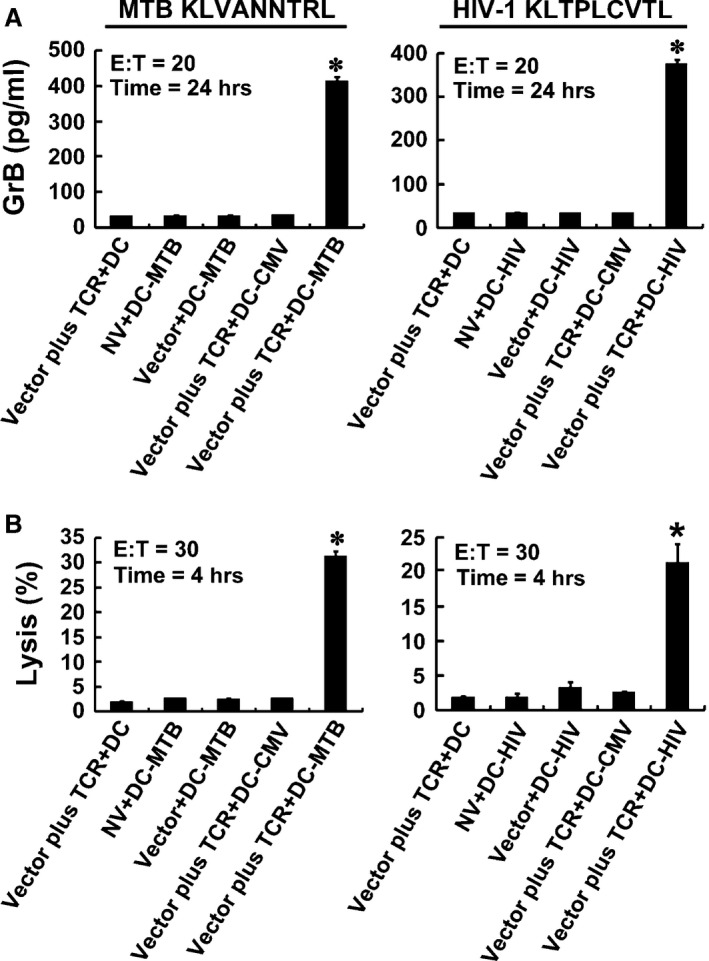
Cytotoxicity of TCR gene‐modified CD8^+^ T cells in response to antigen peptides stimulation. CD8^+^ T cells were cocultured with DCs loaded with 10 μg/ml of MTB Ag85B_199‐207_ (KLVANNTRL) (left panel) or HIV‐1 Env_120‐128_ (KLTPLCVTL) (right panel). Secretion of GrB by T cells was assayed using ELISA (**A**), and the direct cytotoxicity on target cells was analysed using DELFIA assay (**B**).*P < 0.05 compared with NV + DC‐MTB group or NV + DC‐HIV group.

Using as target cells, DCs loaded with MTB Ag85B_199‐207_ peptide or HIV‐1 Env_120‐128_ peptide were incubated with the TCR gene‐modified T cells, and GrB secretion and the percent‐specific lysis were significantly higher than that in the untransduced or empty vector‐transduced T cells (*P* < 0.05). However, there was no significant difference between the unloaded group and the CMV pp65_495‐503_‐loaded group (*P* > 0.05). The results showed that the TCR gene‐modified T cells possessed specific cytolytic activity against MTB/HIV‐1 peptides at the same time (Fig. 5).

### Activities of TCR gene‐modified CD8^+^ T cells against endogenous antigens

**Figure 6:**
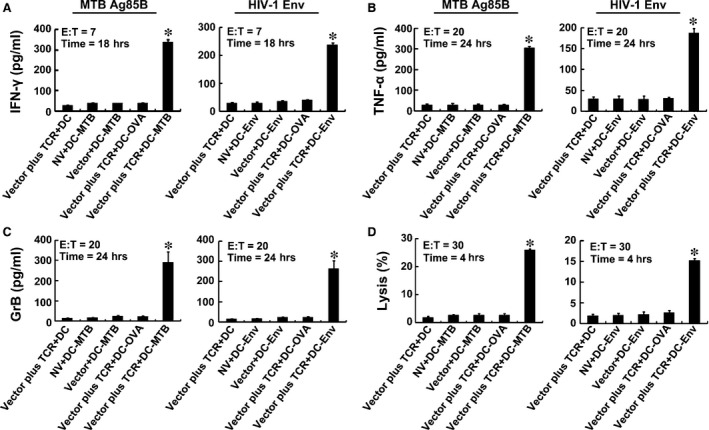
Immune responses to endogenous antigens by TCR gene‐modified CD8^+^ T cells. Activities of T cells were analysed by ELISA of IFN‐γ (**A**), TNF‐α (**B**), GrB (**C**) and DELFIA assay (**D**). DC‐MTB, DC‐Env, DC‐OVA: DCs transfected with the Ag85B‐, Env‐ or OVA‐expressing plasmid. *P < 0.05 compared with NV + DC‐MTB group or NV + DC‐Env group.

Dendritic cells transfected with the Ag85B‐expressing plasmid pV1J.ns‐tPA‐Ag85B, the Env‐expressing plasmid pCAGGS‐Env or the OVA‐expressing plasmid pCI‐OVA were cocultured with TCR gene‐modified CD8^+^ T cells to further identify the activity of TCR gene‐modified T cells against the intracellular infectious pathogens. In line with expectations, the TCR gene‐modified CD8^+^ T cells responded to both Ag85B‐ and Env‐expressing plasmid with significantly higher levels of cytokine secretion and cytotoxic function when compared with T cells without TCR gene modification or without specific antigen presentation (*P* < 0.05). These results demonstrated that the TCR gene‐modified CD8^+^ T cells against endogenous antigens were functional and peptide specific (Fig. 6).

### CD69 expression in TCR‐transduced J.RT3‐T3.5 cells

**Figure 7:**
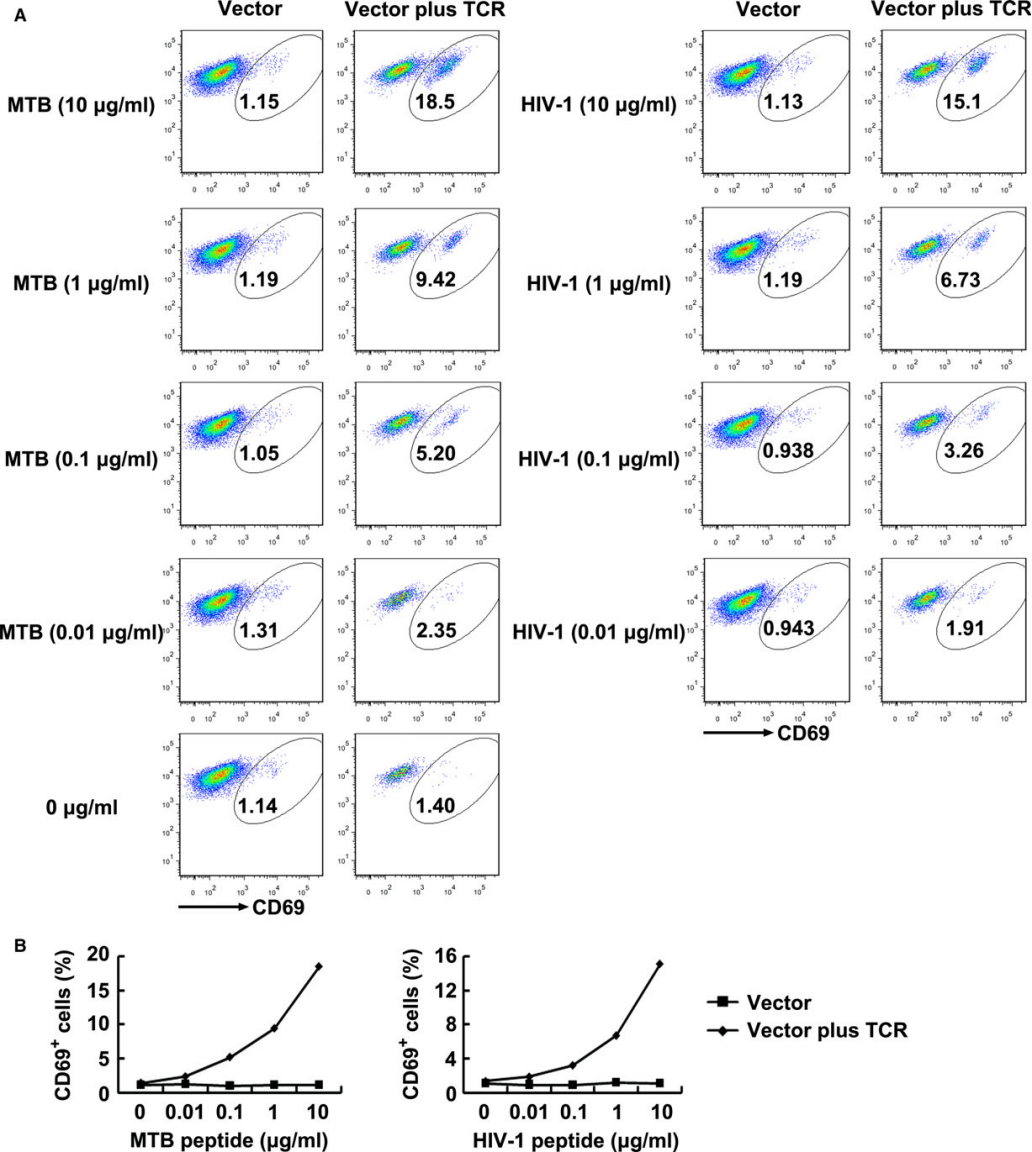
CD69 expression of TCR‐transduced J.RT3‐T3.5 cells in response to antigen peptides stimulation. (**A**) Shown are the resultant FACS scatter plots for CD69 expression on J.RT3‐T3.5 cells transduced with empty vector or TCR vector within the GFP‐positive population. Cells were cocultured with T2 cells pulsed with serial dilutions of Ag85B_199‐207_ or Env_120‐128_ peptide. (**B**) Shown are the line charts analysed for the FACS scatter plots. MTB peptide: Ag85B_199‐207_ peptide; HIV‐1 peptide: Env_120‐128_ peptide. Vector: J.RT3‐T3.5 cells transduced with empty vector; Vector plus TCR: J.RT3‐T3.5 cells transduced with TCR vector.

To further demonstrate that the bispecificity of the TCR was not achieved *via* misparing with other TCR chains in the polyclonal CD8^+^ T cells, the TCR gene was transfected into the TCR negative T cell line J.RT3‐T3.5. Using as antigen‐presenting cells, T2 cells loaded with serial dilutions of the MTB Ag85B_199‐207_ peptide or HIV‐1 Env_120‐128_ peptide were incubated with the TCR‐transduced J.RT3‐T3.5 cells. As expected, the early activation marker CD69 expression of the modified J.RT3‐T3.5 cells responded to both peptides can be detected at peptide concentrations as low as 0.01 μg/ml and increased in a dose‐dependent manner. Comparably, no significant increase in CD69 expression was observed in empty vector‐transduced cells at every peptide concentration (Fig. 7).

## Discussion

In this study, a single TCR specific for both MTB peptide Ag85B_199‐207_ and HIV‐1 peptide Env_120‐128_ was screened out from PBMCs of a HLA‐A*0201^+^ healthy individual, using the CDR3 spectratype analysis technique with a high degree of practicality and reliability 36, 37. The resulting TCR gene‐modified CD8^+^ T cells showed elevated secretion of IFN‐γ, TNF‐α, GrB and specific cytolytic activity after antigen presentation of either Ag85B_199‐207_ or Env_120‐128_ by autologous DCs *in vitro*, indicating that the TCR gene‐modified CD8^+^ T cells possessed activity of responding to two different antigenic peptides simultaneously.

The two different pathogens, MTB and HIV‐1, are all intracellular pathogens that are mainly controlled by effective T cell‐mediated immune responses. Although mainly showing a proinflammatory instead of anti‐inflammatory cytokine profile 38, MTB/HIV‐1 coinfection is principally featured as immunosuppression due to CD4^+^ T cell depletion and subsequent reduction in antigen‐specific cytokine responses 39. Cells expressing a specific set of cytokines such as IFN‐γ, IL‐2, TNF and/or IL‐17 are present in quite reduced numbers in MTB/HIV‐1 coinfected patients 40, subsequently resulting in significant weakness of critical protective immune responses. Therefore, adoptive transfer of a multitude of antigen‐specific CTLs to coinfected patients may enhance their cellular immune function.

It is worth noting that HIV‐1 can enter a state of latency that allows it to persist for decades in ART‐treated patients so that there are many challenges for T cell–mediated elimination of HIV reservoirs 41. In a recent article, Deng *et al*. 42 have demonstrated that CTL escape variants of HIV‐1 completely dominate the latent viral reservoir, presenting another challenge to HIV‐1 cure efforts. However, in chronically infected patients, after latency is reversed, CTL clones only targeting different unmutated epitopes, rather than CTL‐escaped epitopes, can efficiently recognize and eliminate autologous CD4^+^ T cells infected with replication‐competent virus derived from the latent reservoir, despite the presence of immunodominant CTL escape mutations in some HIV‐1 epitopes. Therefore, directing broader CTL responses to unmutated viral epitopes is indispensable to clear latent HIV‐1. It is important to note that MTB, which is similar to HIV‐1, also can enter a latent state and accumulates mutations during latency, which render adaptive immune responses incapable of eliminating MTB completely 43. The research idea of HIV‐1 may also be appropriate for treatment of MTB infection. Consequently, the use of genetically engineered CTLs that are pre‐programmed with native (unmodified) TCRs, affinity‐enhanced TCRs, or ‘chimeric’ antigen receptors 44 specific for these alternative epitopes might also prove useful at this point. In our study, the bispecific TCR against highly conserved MTB and HIV‐1 epitopes engineered CTLs may become a vital member of numerous sweepers which can eliminate MTB and HIV‐1.

There may be three types of strategies of adoptive cellular immunotherapy for MTB/HIV‐1 coinfection. First, two groups of T cells transduced with the MTB‐specific TCR and the HIV‐specific TCR, respectively, are reinfused collaboratively into MTB/HIV‐1 coinfection patients. Second, the MTB‐specific TCR and HIV‐specific TCR are transduced to the same T cell population, which is then given the dual specificity and infused into coinfection individuals. Third, find a single TCR bispecific for both MTB and HIV‐1 to transduce T cells followed by adoptive transfer.

The technology used in the first strategy is relatively easy to operate, but accompanied with two main flaws about number and function. First, when adoptively transfer the same number of T cells, infusing mixture of two kinds of antigen‐specific T cells immediately loses the quantity advantage compared with one kind. Although there are two antigenic specificities, immune responses against each pathogen may be insufficient. Second, due to the limitations of the current technology, MTB‐ and HIV‐specific T cells cannot selectively migrate to the TB lesions or HIV‐infected sites, respectively, to play a role on both pathogens simultaneously. It may occur that MTB‐specific T cells migrate to the HIV‐infected sites so that they could not play their correct immune effects. The same is the case with HIV‐specific T cells. For the second strategy, transduction of MTB‐specific TCR and HIV‐specific TCR to the same T cell population could realize dual specificity at the single cell level, but strong competitive effects may occur at the same time, resulting in reduced expression of both two exogenous TCRs. Hofmann *et al*. 45 have reported that after both HIV gagTCR and nefTCR were transduced to the same T cells simultaneously, the T cells diminished their reactivity to either gag or nef epitopes compared with transduction of one TCR alone. The co‐expression of two additional TCRs in one cell is not expected given the assumption that either TCR can signal independently and rather indicates that some mechanisms of competition or mutual interference exist 45. However, this problem can be resolved at least in part using adapted amounts of the human gagTCR and the CD3 binding of the murinized nefTCR 45. The third strategy makes up for the defects of above two strategies. Meanwhile, the technology is relatively simple. Hence, we expected to find a single TCR bispecific for both MTB and HIV‐1 antigen peptides from TCR repertoire of healthy people to endow primary T cells with activities of anti‐MTB/HIV‐1 antigen peptides *in vitro*, which provide a novel means for the immunotherapy of MTB/HIV‐1 coinfected patients.

To provide effective immune coverage, a single TCR must recognize over one million different peptides in the context of a single MHC class I molecule, which is called as T cell cross‐reactivity 24, 46. The idea that immune coverage is provided by limited quantity of hugely cross‐reactive T cells possesses many positive consequences 23. First, because far fewer T cells are needed to scan any infected cell than suggested by the clonal selection theory which strictly upholds ‘one‐clonotype‐one‐specificity’, a system with limited numbers of highly cross‐reactive T cells is both temporally and spatially beneficial. Second, widespread T cell cross‐reactivity provides remarkable conservation of resources by generating ‘one lamp with several switches’. It is well known that T cell cross‐reactivity among more than one infection is referred to as heterologous immunity 47. Heterologous immunity between related pathogens is common. Indeed, the presence of extensive T cell cross‐reactivity means that heterologous immunity can not only extend beyond the cross‐recognition of pathogens with high sequence similarity but also to very dissimilar antigens. For example, cells that are specific for the immunodominant GILGFVFTL peptide from influenza virus can often recognize the Epstein–Barr virus epitope GLCTLVAML 48 or the immunodominant HIV‐derived SLYNTVATL antigen 49 (all of which are HLA‐A2 restricted). For these reason, to find a cross‐reactive TCR for engineering CD8^+^ T cells in order to provide advanced treatment for MTB/HIV‐1 coinfected persons is practicable. Indeed, in this study, we found a cross‐reactive TCR recognizing dissimilar MTB Ag85B_199‐207_ (KLVANNTRL) and HIV‐1 Env_120‐128_ (KLTPLCVTL) epitopes.

The proteins of the antigen 85 (Ag85) complex consists of three internally cross‐reacting antigens encoded by three genes located at separate sites in the mycobacterial genome and represent the quantitatively major part of the secreted proteins constituent of mycobacterial culture fluids 50, 51, 52. Each protein of Ag85 complex acts as a mycolyltransferase involved in the final stages of mycobacterial cell wall assembly 53. Among them, Ag85B is the most abundant secreted protein after exposure to MTB and could induce highly significant protective immunity 54, 55, 56. The Ag85B_199‐207_ (KLVANNTRL) epitope that is strongly recognized by HLA‐A*0201‐restricted CD8^+^ T cells in HLA‐transgenic mice and humans is highly conserved. CD8^+^, HLA‐A*0201‐restricted T cells directed against Ag85B_199‐207_ peptide are present in the human T cell repertoire, producing high levers of IFN‐γ and TNF‐α and effectively recognizing *Mycobacterium*‐infected macrophages 57. Similarly, the envelope protein gp160 of HIV‐1 also can provide immune protection. The early inhibition of HIV replication appears to depend on the development of HIV‐specific CTL responses against numerous viral proteins, including the envelope protein gp160 58, 59. The HIV‐1 Env_120‐128_ (KLTPLCVTL) peptide locating in the N‐terminal conserved regions of gp160 protein is highly conserved among HIV‐1 strains of the B subtype 60, 61, presenting in 80% of the variant forms of the HIV‐1 peptide sequences and inducing high levels of IFN‐γ in human HLA‐A*0201^+^ donors 62. CTL activity derived from PBMC of the HIV‐1‐infected individuals stimulated *in vitro* with peptide was demonstrated against at least two novel minimal env‐encoded conserved epitopes, and Env_120‐128_ is one of them 60. It is noteworthy that Position 1 (P1), P2 and P9 AAs of Ag85B_199‐207_ and Env_120‐128_ are exactly in common, with two of them important for binding to the MHC molecular. In this case, a TCR specific for both the two antigenic peptides is reasonably existed.

In summary, we used TCR gene modification to obtain a large number of dual‐specific T cells which were confirmed to possess enhanced antigen peptide‐specific activity. To the best of our knowledge, this article is first proposed to produce responses against two dissimilar antigenic peptides of MTB and HIV‐1 simultaneously by transducing CD8^+^ T cells with a single TCR. This strategy might be useful in immunotherapy for MTB/HIV‐1 coinfected individuals.

## Conflict of interest

There is no potential conflict of interest.

## Author contribution

CZ participated in the cell experiments, substantially contributed to molecular biology studies and immunoassays, performed the statistical analysis and drafted the manuscript. QW participated in research design, interpretation of data and molecular biology studies. XC participated in research design, cell and virus infection experiments and molecular biology studies. RW participated in cell and virus infection experiments and molecular biology studies. WH participated in cell and virus infection experiments. SZ participated in cell experiments and molecular biology studies. XD participated in cell culture, immunoassay and interpretation of data. LM conceived of the study, participated in its design and coordination and revised the article critically. All authors read and approved the final manuscript.
